# Long-term effects of chronic stress models in adult mice

**DOI:** 10.1007/s00702-023-02598-6

**Published:** 2023-02-14

**Authors:** Inès Tran, Anne-Kathrin Gellner

**Affiliations:** 1 Institute of Physiology II, Medical Faculty, University of Bonn, Bonn, Germany; 2 Department of Psychiatry and Psychotherapy, University Hospital Bonn, Bonn, Germany

**Keywords:** Preclinical models, Chronic disease, Neuropsychiatric conditions, Mouse models, Depression, Anxiety, Stress-induced, Stress-associated, Post-traumatic stress

## Abstract

Neuropsychiatric disorders, such as major depression, anxiety disorders, and post-traumatic stress disorder, tend to be long-term conditions in whose development and maintenance stress are central pathogenic factors. Translational mouse models are widely used in neuropsychiatric research, exploiting social and non-social stressors to investigate the mechanisms underlying their detrimental effects. However, most studies focus on the short-term consequences of chronic stress, whereas only a few are interested in the long-term course. This is counterintuitive given the human conditions that preclinical models are designed to mimic. In this review, we have summarized the limited work to date on long-term effects of chronic stress in mice models. First, the different models are presented and a definition of short- vs. long-term sequelae is proposed. On this basis, behavioral, endocrine, and vegetative effects are addressed before examining data on cellular and molecular alterations in the brain. Finally, future directions for research on the long-term effects of stress are discussed.

## Introduction

Stress is commonly referred to as a stimulus, or stressor, that disrupts the physiological homeostasis of the organism. Whether a stressed individual develops adaptive or maladaptive consequences depends on the severity of the stimulus, but also on the ability to cope at the behavioral, cellular, and molecular level.

In addition to the intensity of a stressor, the time course of stress is highly relevant for its pathogenic effects. There is no clear definition of chronic stress in humans, but typically it is a repeated exposure to physical and very often social stressors that can last for years (Kessler et al. 2005). In translational animal models, chronic stress is typically exerted over a period of at least 10 days and can last up to several weeks, resulting in long-lasting changes of behavior and physiology (Venzala et al. 2013; Ménard et al. 2016; Koolhaas et al. 2017).

Repeated, chronic stress exposure is a known risk factor for the development of neuropsychiatric disorders in humans, including major depression, post-traumatic stress disorder, co-morbid anxiety (Kessler 1997; Kessler et al. 2005; Ménard et al. 2016; Davis et al. 2017), and neurodegenerative diseases (Swaab et al. 2005). Moreover, stress can also lead to systemic diseases outside the central nervous system, such as those of the cardiovascular system (Esch et al. 2002; Steptoe and Kivimäki 2012). All of these disorders and symptoms are known to be long-lasting, even with appropriate therapies (Fava and Visani 2008). The brain is an important target of stress, and it responds to psychological or physical stressors with emotional, behavioral, cellular, and molecular adaptations, as previously shown in rodent (McEwen 2007; Maniam and Morris 2012; McEwen et al. 2015) and human studies (Dai et al. 2020). Stress-related conditions also include alterations of peripheral organs and their physiology. In both rodents and humans, the developmental stage at which the stressor occurs has been shown to determine the timing of onset, type, and duration of stress effects (Lupien et al. 2009). Although stress in the developmental stages prior to adulthood is a major contributor to neuropsychiatric disorders, it is difficult to separate the effects of early and later stressors in humans. Our review focuses on long-term effects after chronic stress during adulthood in mice, a developmental stage when depressive and anxiety disorders occur frequently in humans (Solmi et al. 2022).

Stress duration and persistence of stress effects are related but must be considered independently, especially when terms, such as “long-term” and “chronic”, are used to describe stressors and their consequences. Stressors applied chronically or acutely can lead to permanent adaptations in humans (Davis et al. 2017) and animals (Harbuz and Lightman 1992). Chronic stress in rodent models has been validated to mimic the development of stress-related diseases in humans, considering the limitations of transferring behavior and biology from animals to humans (Willner 1997; Belzung and Lemoine 2011; Planchez et al. 2019).

There is no clear definition or consensus for the end of short-term and the onset of long-term effects following the end of a stressor in rodent models. In most studies, the observation of stress effects is limited to a period immediately after stress up to a few days (Hayashi et al. 2014; Higashida et al. 2018; Borrow et al. 2019), which likely reflects the acute stress response manifesting within one to two weeks in humans. We defined a minimum of one week post stress after which effects had to be reported in mice to be included in this review, although we are aware of the difficulties in translating the rate of aging from mice to humans (Dutta and Sengupta 2016). Nonetheless, our definition for mice should fall within the diagnostic time criterion for depression in humans, in which symptoms must persist for at least two weeks (American Psychiatric Association 2013) before they are considered a manifest clinical condition. Hence, long-term changes in mice in adult paradigms must persist for weeks and even months, which has been observed repeatedly (Avgustinovich et al. 2005; Colyn et al. 2019; Gellner et al. 2022; Okamura et al. 2022). Importantly, symptoms may occur with different latencies post stress in mice and humans, and thus both the onset and duration of stress-related effects need to be examined.

The characteristics of neuropsychiatric disorders in humans to be mimicked in chronic stress models in rodents require the investigation of the entire time course of the various sequelae following chronic stress. Yet, most studies applying chronic stress in rodents still focus on short-term effects (Antoniuk et al. 2019). While the long-term effects of chronic stress in rats have already been highlighted (Buwalda et al. 2005), few studies have focused on or additionally investigated the long-term effects of chronic stress in mice. This is a relevant gap, as chronic behavioral stress paradigms are nowadays predominantly established in mice due to the characteristics of this species, but also due to the greater availability of genetic models and other advanced methods in mice (Ellenbroek and Youn 2016).

The aim of this review is to provide a comprehensive overview of the current knowledge on long-term effects persisting or occurring at least one week after chronic stress in mice. This could ultimately promote and guide future research to translate even more valuable findings from “mice to men”.

## Chronic stress models

Murine chronic behavioral stress models can be divided into those with predominantly social stressors versus those that relying on non-social aversive stimuli, or even a combination of both.

Sociability and social interactions are hallmarks and a prerequisite for healthy life conditions in rodents. Thus, perturbations of social grouping and hierarchical orders are used for various ways of stress induction (Komori et al. 2019; Gellner et al. 2021). It should be noted that the widely studied house mouse (*Mus musculus)* has certain deficits in terms of social bonds and stable pairings observed in humans that are found in less commonly studied rodents such as California mouse or Prairie vole (Beery et al. 2018; Lee and Beery 2019).

In general, social interaction can be a negative source of stress or a positive resource, depending on the type of interaction and individual characteristics in mice and humans (Wood and Bhatnagar 2015). The chronic social defeat stress model (CSDS) uses strong physical attacks from a dominant, larger male (aggressor) on a smaller male that invades the home cage of a new resident mouse daily, typically 10 days in a row (Golden et al. 2011; Hollis and Kabbaj 2014). This paradigm is widely used and considered as an etiologically valid model for depression and anxiety disorders because it produces robust phenotypes of stress-susceptible and -resistant mice (Avgustinovich et al. 2005; Berton et al. 2006; Krishnan et al. 2007).

Another psychosocial stress model for male mice is the Chronic Subordinate Colony Housing Paradigm (CSC). It consists of housing four male experimental mice with a larger dominant male for 19 consecutive days, with the dominant male replaced on days eight and 15 to avoid habituation (Reber et al. 2007). This paradigm has also shown high validity in mimicking human stress-induced diseases and outcomes (Langgartner et al. 2015). From the same group that developed the CSC model, another social stress paradigm called social defeat/overcrowding (SD/OC) has been established in males, also lasting 19 days: here, animals are exposed to social defeat (2 h) or co-housing with a large group of mice on different days throughout the stress period (for details see Reber et al. 2006).

Other social stress paradigms, such as social isolation, maternal separation, or prenatal stress (McEwen 2007; Becker et al. 2021), are applied at a pre-adult stage or have not yet been studied for long-term effects and are therefore not included in this review.

Models with non-social stressors have also been established to study the pathogenesis of stress-related diseases (Planchez et al. 2019; Becker et al. 2021). Along with CSDS, chronic restraint stress (CRS) is one of the most commonly used models of chronic stress in mice. Protocols can vary widely, but typically the test mouse is immobilized in a bag or container for several hours daily, repeated for up to four weeks (Kim and Han 2006; Mao et al. 2022). Another common non-social model is chronic unpredictable mild stress (called CUMS or CMS). It combines various aversive physical stimuli, such as immobilization, electric shocks, tail suspension, forced swimming, orbital shaking, tilting the cage 45°, wet bedding, empty cage, noise stress, flashlight, light/dark change, and water/food deprivation, over a period of at least three weeks (Zhao et al. 2012; Erburu et al. 2015; Antoniuk et al. 2019; Picard et al. 2021). Although there are social stimuli in some CUMS models, such as the presentation of bedding or feces from other mice or rats, we consider CUMS as a non-social model in contrast to CSDS.

While non-social stress models can be applied to both sexes, the psychosocial stress paradigms described above and widely used in research are limited to male mice. Given that effective variations of social defeat stress have recently been reported in female mice (Laredo et al. 2015; Newman et al. 2019), future studies should not fail to investigate their long-term outcomes. Alternative models have been developed for females: Furman and colleagues used social crowding with CD-1 mice compared to classical CSDS in males and subsequently reported long-lasting stress effects (Furman et al. 2022).

First, we will summarize the behavioral outcomes including cognition, then endocrine and vegetative effects. In the second half, we will focus on the central effects in the brain, including structural and molecular changes.

## Behavioral effects

Chronic stress is typically associated with symptoms of anxiety and depression, such as anhedonia, social withdrawal, and reduced self-care in mice, and can be viewed as mimicking human clinical symptoms (Kessler 1997; Willner 1997; Belzung and Lemoine 2011; Davis et al. 2017; Becker et al. 2021). For brevity and focus, we will only briefly describe the behavioral paradigms used to investigate mouse behavior, while an overview of them can be found elsewhere (Planchez et al. 2019; Becker et al. 2021).

### Social behavior

Social withdrawal is a hallmark of CSDS and is subsequently assessed using the social avoidance test. For this, the mouse is first presented with an empty wire cage in an open field arena before an unknown aggressor mouse is placed in this cage. The time the mouse interacts with the empty cage compared to the filled cage is measured, and usually, a ratio or the absolute social interaction time is compared to define social avoidance (Golden et al. 2011). This behavior is thought to be closely related to social withdrawal in depressed humans (Komori et al. 2019; Gellner et al. 2021). CSDS leads to the development of social avoidance in many mice that are naturally high in sociability. Impairment of this natural interest has been shown to persist for one week up to more than four weeks after cessation of CSDS (Berton et al. 2006; Tsankova et al. 2006; Krishnan et al. 2007; Razzoli et al. 2011d; Trainor et al. 2011; Venzala et al. 2013; Warren et al. 2013; Colyn et al. 2019; Lehmann et al. 2019; Gellner et al. 2022; Okamura et al. 2022). Importantly, one study revealed that long-term social avoidance after CSDS depends on the mouse strain and persists in BalbC but not in the more commonly used C57BL6/J mice (Razzoli et al. 2011c). It was previously assumed that social avoidance behavior occurs in the vast majority (up to 70%) of mice after CSDS (Golden et al. 2011) and that this is the gold standard for defining stress susceptibility and resilience in this model. This approach was recently challenged by demonstrating the non-correlation of social avoidance and anhedonia in adolescent mice in the short term (Alves-dos-Santos et al. 2020), which we confirmed in adult mice in the long and short term. To this end, we recently introduced a multimodal behavioral assessment based on the joint scoring of multiple tests to characterize individual stress susceptibility both short and long term after CSDS (Gellner et al. 2022; Serradas et al. 2022). Moreover, Okamura and colleagues have demonstrated the differential time course of social avoidance and anxiety symptoms following defeat stress (Okamura et al. 2022). Interestingly, although social avoidance was not found in CSC or SD/OC mice one week post stress, interaction with an empty cage was reduced and may indicate anxiety-like behavior toward a novel object (Slattery et al. 2012).

Social behavior is also tested after non-social stress like CUMS, and reduced sociability in the social interaction test was reported two weeks post stress (Erburu et al. 2015).

### Anxiety-like behavior

A series of tests is used to detect signs of anxiety in mice following chronic stress. These tests involve balancing the natural urge to explore new things against avoidance of threatening situations, such as unprotected and unsheltered locations, bright lighting, or heights (Crawley and Bailey 2008).

Several studies revealed a long-term increase in anxiety following chronic stress, resulting in animals spending less time in the open zones in the elevated plus maze (EPM) or increased latency to enter the zones. For social stressors, this anxiety-related behavior has been reported between one week up to a month after CSDS (Avgustinovich et al. 2005; Venzala et al. 2013; Warren et al. 2013; Okamura et al. 2022) and one week after CSC or SD/OC (Slattery et al. 2012). For the non-social CUMS model, this was found up to one month after exposure (Erburu et al. 2015). For both CSDS (Avgustinovich et al. 2005; Wohleb et al. 2014) and CRS (Khalid et al. 2016), it was reported after one to two weeks that they spent less time in the unprotected center of an open field. For CSDS, this effect had disappeared at day 24 (Wohleb et al. 2014) and for CRS three weeks post stress (Khalid et al. 2016). Venzala and colleagues also measured the time spent in the eating zone in the middle of an open field and the latency to approach it in the novelty-suppressed feeding test (NSF) (Venzala et al. 2013). Consistent with their other results, the time in the eating zone was lower and latency higher after CSDS at four weeks post exposure. This suggests a higher level of long-term anxiety, even in the face of prior food deprivation (Venzala et al. 2013). At 20 days after CRS (Chotiwat and Harris 2006) and one month after CSDS (Lehmann et al. 2019), a decreased time spent in the light zone of the Light and Dark Box Test (LDT) was found, while another study found no significant differences between CMS mice and control mice at one month (Elizalde et al. 2008). While these results were obtained in male mice, it should be emphasized that females surprisingly spent more time in the light zone one week after the last stress session with social crowding (Furman et al. 2022). Usually, reduced movement and exploration behavior is associated with increased anxiety. On the other hand, it can also be interpreted as a sign of decreased motivation, as these tests induce a conflict between the rodent’s innate instinct to explore new things and aversive stimuli such as bright light (Bourin and Hascoët, 2003). Nevertheless, Furman and colleagues assessed an increase in average velocity during the Light and Dark Box Test a week following social crowding stress in female mice, leading to the interpretation as hyperactive anxious behavior (Furman et al. 2022). Stress-induced hyper-locomotion was also demonstrated in male mice by Venzala and colleagues in the open field test four weeks post CSDS (Venzala et al. 2013), and by Slattery and colleagues for home cage activity one week post CSC (Slattery et al. 2012). Altered motivational drive reflects one of the key symptoms of depression-like behaviors in humans, and the next section addresses further considerations of this in mouse behavior.

### Depression-like behavior

An important purpose of inducing chronic stress in animals is to investigate depression-like behaviors that routinely occur in patients. These behaviors include reduced motivation and self-care, anhedonia, increased despair, and helplessness.

In the forced swim test (FST), increased immobility time is interpreted as a sign of despair and helplessness (Castagné et al. 2011), which has been observed one or two weeks after CSDS (Avgustinovich et al. 2005) and can last up to one month (Warren et al. 2013). Regarding non-social stress, CRS combined with water immersion (WIRS) for three weeks led to increased immobility one week later (Yasugaki et al. 2019). Kim and colleagues introduced another non-social model of chronic stress, termed “emotional stress”, using a water bucket stress paradigm (mice standing on a platform in water for 2 h per day for 14 days). They asked whether this treatment could induce long-lasting depression-like behavior, as previously observed in CRS of the same duration (Kim and Han 2006). They found increased immobility in the FST two weeks following the last water bucket exposure, an effect that persisted for three weeks. This finding is underscored by the fact that the tail suspension test (TST), another indicator of helplessness, also showed increased immobility three weeks after the water bucket stress (Kim et al. 2012). Similar results in the FST or TST paradigm were observed by other groups one week post CSDS (Avgustinovich et al. 2005; Chen et al. 2016), one week post CRS (Khalid et al. 2016) or CMS (Zhao et al. 2012), two weeks post CRS (Kim and Han 2006), and a month post CUMS (Elizalde et al. 2008; Venzala et al. 2013). However, not all studies point in the same direction regarding long-term signs of despair: no effects were found in the FST and/or TST three to four weeks after CSDS in males (Krishnan et al. 2007; Furman et al. 2022) or after chronic social crowding stress in females (Furman et al. 2022).

One of the main symptoms in patients with major depression is anhedonia, i.e., the inability to experience pleasure in activities that are usually considered pleasurable (Willner 1997; Belzung and Lemoine 2011). The sucrose preference test is commonly used to assess this symptom in rodents by offering both water and a sweet solution, the latter usually being the preferred choice (Liu et al. 2018). Some authors reported a reduced preference for sucrose solution that can last up to a month after exposure to social stress (Venzala et al. 2013; Warren et al. 2013; Chen et al. 2016), while this test was inconclusive for others (Krishnan et al. 2007; Gellner et al. 2022). For non-social stress, the literature reports anhedonia one week after WIRS (Yasugaki et al. 2019) and one month after the CUMS model (Elizalde et al. 2008; Venzala et al. 2013; Erburu et al. 2015).

Self-care is also a physiological behavior that can serve as a countermeasure for stress, but this ability is often found to be reduced in depressed individuals. In mice, this ability can be assessed by observing nest-building or grooming behavior. Using the nestlet shredding test (Deacon 2006), we recently reported significantly reduced nest-building behavior two to three weeks after CSDS (Gellner et al. 2022; Serradas et al. 2022). Notably, nest-building was the most robust long-term discriminator between stress-susceptible mice and resilient mice and controls in our studies, along with social avoidance behavior. Finally, Avgustinovich and colleagues described prolonged grooming activity one week after stress (Avgustinovich et al. 2005), a behavior that also reduces stress levels in animals (Spruijt et al. 1992).

### Cognition and learning

Cognitive impairment and impaired learning are frequently associated with stress-associated disorders in humans and have also been studied in the relevant animal models (Keeler and Robbins 2011; Yu et al. 2011). Importantly, impaired fear extinction, as a type of associative learning, is closely associated with post-traumatic stress symptoms in humans (Rothbaum and Davis 2003).

While spatial memory remained intact two weeks after CSDS in the Morris Water Maze test, the same authors found that defeated mice exhibited higher freezing time after contextual fear conditioning than the control group (Yu et al. 2011). Furman and colleagues investigated fear-conditioned memory five to six weeks after social stress and observed enhanced memory retrieval in males but not females, suggesting higher resilience in the latter (Furman et al. 2022).

The novel object recognition test also investigates learning and memory, which was impaired between one week up to one month after the end of CUMS in several studies (Elizalde et al. 2008; Venzala et al. 2013; Erburu et al. 2015). Recently, we reported a pronounced learning deficit on a fine motor task 2.5 weeks after CSDS along with other motor system alterations (Gellner et al. 2022), an area not routinely examined in stress disorders but increasingly recognized to have stress-related impairments (Sabbe et al. 1996; Mergl et al. 2007; Bennabi et al. 2013).

Table 1 provides a summary of the long-term behavioral outcomes related to stress models, post-stress latency, and the behavioral domain.

**Table 1:** Behavioral long-term effects after chronic stress

	Publication	Stress model	Time post-stress	Strain and sex	Social behavior	Anxiety-like behavior	Despair and helplessness	Anhedonia	Self—care	Cognition
Social stress	Avgustinovich et al. (2005)	Partition test + CSDS (30 days)	1 and 2 weeks	C57BL/6 J M		EPM, OFT Parameter: time and entry in OA/center ▼	FST Parameter: immobility time ▲		OFT Parameter: frequency and duration of grooming ▲	
	Berton et al. (2006)	CSDS (10 min/10 days)	4 weeks	Transgenic mice M	Social avoidance test Parameter: IT ▼					
	Colyn et al. (2019)	CSDS (10 min/10 days)	1 month	C57BL/6 J M	Social avoidance test Parameter: IT ▼					
	Furman et al. (2022)	CSS (1 h/15 days)	1–4 weeks	C57BL/6 J M and F		ASR, Parameter: startle response **NS**, LDT (1 wps), Parameter: time in light ▲ in F	FST Parameter: immobility time **NS**			FC Parameter: freezing in M ▲
	Gellner et al. (2022)	CSDS (5 min/10 days)	3 weeks	Thy1-GFP M	Social avoidance test Parameter: IT ▼			SPT Parameter: sucrose preference **NS**	NST Parameter: nest building ▼	Forelimb reaching task, Parameter: fine motor skill learning ▼
	Henderson et al. (2017)	CSDS (10 min/10 days)	1 week	C57BL/6 J M	Social avoidance test Parameter: IT ▼					
	Krishnan et al. (2007)	CSDS (10 min/10 days)	4 weeks	C57BL/6 J M	Social avoidance test Parameter: IT ▼	EPM Parameter: time in OA ▼	FST, TST Parameter: immobility time **NS**	SPT Parameter: sucrose preference **NS**		
	Lehmann et al. (2019)	CSDS (5 min/14 days)	4 weeks	C57BL/6 J M	Social avoidance test Parameter: IT ▼	LDT Parameter: time in light ▼				
	Okamura et al. (2022)	CSDS (10 min/7 days)	2 weeks	Balb/c M	Social avoidance test Parameter: IT ▼	EPM Parameter: time in OA ▼				
	Razzoli et al. (2011a)	CSDS (10 min/10 days)	3 weeks	C57BL/6 J M	Social avoidance test Parameter: IT ▼					
	Razzoli et al. (2011c)	CSDS (10 min/10 days)	4 weeks	C57BL/6 J Balb/c M	Social avoidance test Parameter: IT Balb/c (▼), C57BL/6 J **NS**					
	Serradas et al. (2022)	CSDS (5 min/10 days)	18 days	C57BL/6 J M					NST Parameter: nest building ▼	
	Slattery et al. (2012)	CSC and SD/OC (19 days)	1 week	C57BL/6 J M	Social avoidance test Parameter: IT with mouse **NS,** with empty cage ▼	EPM Parameter: time in OA▼ Home cage activity Parameter: locomotion▲				
	Trainor et al. (2011)	CSDS (7 min/3 days or 10 attacks)	> 1 month	California mice M and F	Social avoidance test Parameter: IT ▼	OFT, Parameter: time in center **NS** LDT, Parameter: time in light F ▲, M **NS**				
	Tsankova et al. (2006)	CSDS (10 min/10 days)	4 weeks	C57BL/6 J M	Social avoidance test Parameter: IT ▼					
	Venzala et al. (2013)	CSDS (10d), CMS ( 6w)	3.5–4 weeks	C57BL/6 J M	Social avoidance test Parameter: AT ▲	EPM, Parameter: time in OA▼ latency to enter OA ▲ NSF, Parameter: time in eating zone ▼ latency to enter eating zone ▲	FST (only CMS) Parameter: immobility time ▲	SPT Parameter: sucrose preference ▼		NORT Parameter: discrimination of NO ▼
	Warren et al. (2013)	CSDS and “witness “ CSDS (10 min/10 days)	4 weeks	C57BL/6 J M	Social avoidance test Parameter: IT ▼	EPM Parameter: time in OA ▼	FST Parameter: immobility time ▲	Test: SPT Parameter: sucrose preference ▼		
	Wohleb et al. (2014)	CSDS (2 h/6 days)	8–24 days	C57BL/6 J M	Social avoidance test Parameter: IT ▼	OFT (8 dps) Parameter: time in center ▼				
	Yu et al. (2011)	CSDS (10 min/14 days)	2 weeks	C57BL/6 J M						WM, Parameter: time to reach platform **NS** FC, Parameter: freezing ▲
Non-social stress	Chotiwat and Harris (2006)	CRS (2 h/3 days)	12–20 days	NIH swiss M		EPM (12 dps), Parameter: time and entry in OA ▼ LDT(20 dps), Parameter: time in light ▼				
	Erburu et al. (2015)	CUMS (6 weeks)	3–4 weeks	C57BL/6 J M	Social avoidance test Parameter: IT ▼	EPM Parameter: latency to enter OA ▲		SPT Parameter: sucrose preference ▼		NOR Parameter: discrimination of NO ▼
	Elizalde et al. (2008)	CUMS (6 weeks)	1 month	C57BL/6 J M		EPM, Parameter: time and entry in OA **NS** LDT, Parameter: time in light **NS**	FST Parameter: immobility time ▲	SPT Parameter: sucrose preference ▼		NOR Parameter: discrimination of NO ▼
	Khalid et al. (2016)	CRS (4 weeks)	1 and 3 weeks	C57BL/6 J M		OFT (1 wps) Parameter: time in center ▼, distance ▼	FST (1 wps) Parameter: immobility time ▲			
	Kim and Han (2006)	CRS (2–8 h/14 days)	2 weeks	C57BL/6 J M			FST Parameter: immobility time ▲			
	Kim et al. (2012)	Water bucket stress (2 h/14 days)	2–4 weeks	C57BL/6 J M			FST, TST Parameter: immobility time ▲			
	Yasugaki et al. (2019)	WIRS (3 weeks)	1 week	C57BL/6 J M			FST Parameter: immobility time ▲	SPT Parameter: sucrose preference ▼		
	Zhao et al. (2012)	CUMS (7 weeks)	1–3 weeks	C57BL/6 J M			FST, TST (1 wps) Parameter: immobility time ▲			

*ASR* Acoustic Startle Response, *AT* Avoidance Time, *CMS* Chronic Mild Stress, *CRS* Chronic Restraint Stress, *CSC* Chronic Subordinate Colony housing, *CSDS* Chronic Social Defeat Stress, *dps* days post-stress, *EPM* Elevated-Plus-Maze, *F* Females, *FC* Fear Conditioning, *FST* Force Swim Test, *IT* Interaction Time, *LDT* Light and Dark Test , *M* Males, *NO* Novel Object, *NORT* Novel Object Recognition Test, *NS* non-significant, *NST* Nestlet Shredding Test, *OA* Open Arms, *OFT* Open Field Test, *SD/OC* social defeat/ overcrowding, *SPT* Sucrose Preference Test, *TST* Tail Suspension Test, *WM* water maze, *wps* week post-stress, ▲/▼ increase/decrease compared to control or baseline if not stated otherwise, in () when trend

## Endocrine and vegetative effects

### Endocrine changes

Stress sequelae are often divided into those occurring inside and those occurring outside the central nervous system (CNS), although both compartments interact and regulate each other. Nevertheless, to ensure a systematic ordering of the results, we decided to first examine the long-term consequences related to stress hormones and vegetative functions before moving on to the actual outcomes in the brain. The hypothalamic–pituitary–adrenal axis (HPAA) plays a pivotal role in protecting homeostasis during the stress response by modulating functions, such as feeding and reproductive behavior, cognition, emotions, sleep, and temperature. The HPAA is primarily activated when an acute stressor cannot be quickly relieved and becomes a chronic burden (Tsigos and Chrousos 2002). The cascade triggered by stress sends hypothalamic corticotropin-releasing hormone (CRH) to the anterior pituitary gland, which releases adrenocorticotropic hormone (ACTH) into the bloodstream. ACTH in turn induces the production of glucocorticoid hormones [GC, cortisol in humans, and corticosterone (CORT) in rodents] in the adrenal glands and their release into the blood (Oyola and Handa 2017). Glucocorticoids activate the glucocorticoid receptor (GR) inside and outside the CNS, which leads to regulation of physiological and behavioral responses as an adaptation to chronic stress (Harris 2015). When the stressor exceeds the individual’s ability to adapt, physiological GC release is disrupted, which has detrimental effects on the whole organism (Munck et al. 1984; Dai et al. 2020). Corticosterone can be measured acutely or repeatedly in serum or plasma of mice, but also cumulatively in their feces, typically collected in a 24-h period after stress and common in the work of our group (Gellner et al. 2022; Serradas et al. 2022). While our recent studies characterized the acute HPAA response 24 h after the last CSDS in plasma in conjunction with the cumulative release from the first 24 h in feces, we were able to clearly correlate these early increases in plasma CORT and decreased cumulative CORT in feces with an increased adrenal weight seen five weeks after CSDS in stress-susceptible mice (Gellner et al. 2022). A study by Razzoli and colleagues corroborates this long-term finding and showed increased adrenal gland weight five weeks after social defeat (Razzoli et al. 2011d). It should be noted that in other studies of that group, no change in adrenal weight was found at the same time point post stress (Razzoli et al. 2011c; b). From a translational perspective, an enlarged adrenal gland size is consistent with chronically altered HPAA function and can also be found in patients with major depression (Rubin 1995). Krishnan and colleagues found decreased serum CORT levels four weeks post CSDS in susceptible male mice, while levels had increased in resilient males (Krishnan et al. 2007). This could again be the result of a permanently impaired HPAA in susceptible mice as indicated by the adrenal gland weight mentioned above. Interestingly, four weeks after CSDS, increased CORT levels were found in the blood of male but not female California mice (Trainor et al. 2011), whereas another study of C57BL/6 mice two months after chronic social stress paradigms showed a decrease in CORT in male but not female mice (Furman et al. 2022). This may be due to the strains of mice used in these studies having different sex-specific social traits, which also require the development and use of different stress models in females.

Importantly, activation of glucocorticoid receptors can also modify feeding behavior in humans and animals (Maniam and Morris 2012), affecting body composition and weight. Their stress-induced long-term changes are discussed in the next section.

### Weight development and vegetative changes

Chronic stress affects the entire organism and its systems that are necessary for the maintenance of homeostasis. Often the effects are interdependent, such as feeding behavior and weight development or circadian rhythm and sleep. These are also the most important parameters for which long-term data from mice are available.

Chronic stress is usually associated with reduced food intake and weight, due to anhedonia and loss of interest in palatable foods (Maniam and Morris 2012). Hyperphagia and an increase in body weight have been reported for the CSDS model, which has been interpreted as a compensatory mechanism: Razzoli and colleagues demonstrated a steady increase in body weight together with higher food intake between week two and four after CSDS in C57BL6/J compared to the control group, while the BalbC strain lost weight during this period (Razzoli et al. 2011b). This again highlights the importance of long-term observations considering different strains of mice, as to our knowledge such data are not currently available for stress models other than CSDS. With respect to sex, Furman and colleagues found that social stress-induced reductions in body weight in female mice persisted after one week, then returned to control levels and remained there for up to two months (Furman et al. 2022). In the same study, weight loss in males was not different from that of the control group as early as the end of CSDS and for the following five weeks. Other studies also found no significant difference between stressed and control mice at four weeks, despite an initial weight loss during chronic social stress (Krishnan et al. 2007; Warren et al. 2013).

Macroscopic tissue changes serve as indicators of systemic disturbances. One week after CSC, increased adrenal and pituitary weight was noted, but not after SD/OC (Slattery et al. 2012). In another study, decreased spleen, seminal vesicle, and abdominal fat weight were noted one month after the last CSDS session (Razzoli et al. 2011d).

With respect to sleep, CSDS was shown to induce insomnia even three weeks after exposure by increasing daily wake time (Henderson et al. 2017). Similarly, changes in circadian pathways were highlighted one week after CUMS (Erburu et al. 2015).

Finally, cardiac mass and hyperthermia triggered by social interaction were increased four weeks after CSDS (Krishnan et al. 2007).

Overall, further investigation and disaggregation of long-term vegetative stress consequences are clearly needed, as these bodily functions are causally related to non-psychiatric comorbidities, e.g., cardiovascular in humans (Cappuccio et al. 2011; Steptoe and Kivimäki 2012; Powell-Wiley et al. 2021). Table 2 provides a systematic overview of the endocrine and autonomic effects in relation to stress models, post-stress latency, and the parameters studied.

**Table 2:** Endocrine and vegetative long-term effects after chronic stress

	Publication	Stress model	Time post-stress	Strain and sex	CORT level	Body weight	Food/water intake	Sleep disturbance	Organ weights	Temperature/cardiac
Social stress	Furman et al. (2022)	CSS (1 h/15 days)	2 months	C57BL/6 J M and F	Plasma M ▼	F ▼ (1 wps) F **NS** (2 mps)				
	Gellner et al. (2022)	CSDS (5 min/10 days)	5 weeks	Thy1-GFP M					AG ▲	
	Henderson et al. (2017)	CSDS (5 min/10 days)	3 weeks	C57BL/6 J M				Wake time ▲		
	Krishnan et al. (2007)	CSDS (10 min/10 days)	4 weeks	C57BL/6 J M	Plasma ▼	**NS**		**NS**		Social hyperthermia ▲ Cardiac mass ▲
	Razzoli et al. (2011b)	CSDS (10 min/10 days)	1–4 weeks	C57BL/6 J Balb/c M	C57BL/6 J **NS** Balb/c **NS**	C57BL/6 J (2—4 wps) ▲	Food: 1 wps C57BL/6 J **NS,** BALB/c ▲ Food Efficency:1 wps C57BL/6 J ▲, BALB/c **NS**		C57BL/6 J: T, AF ▼ AG, TH, SV **NS** BALB/c: S, AF, SV ▼ AG, TH, T **NS**	
	Razzoli et al. (2011d)	CSDS (10 min/10 days)	4 weeks	TrkB.T1 mice		▼	Food: ▼ Food Efficiency: ▼		AG ▲ S, SV, AF ▼	
	Slattery et al. (2012)	CSC and SD/OC (19 days)	1 week	C57BL/6 J M					CSC: AG, PG ▲ SD/OC: **NS**	
	Trainor et al. (2011)	CSDS (7 min/3 days or 10 attacks)	4 weeks	California mice M and F	Plasma F **NS,** M ▲	▼				
	Warren et al. (2013)	CSDS and “witness “ CSDS (10 min/10 days)	4 weeks	C57BL/6 J M		Stable after 4 weeks				
Non-social stress	Erburu et al. (2015)	CMS (6 weeks)	30 days	C57BL/6 J M		**NS**		circadian rhythm pathway ▼ (1 wps)		
	Kim et al. (2012)	Water bucket stress (2 h/14 days)	> 3 weeks	C57BL/6 J M	Slow return to baseline after stress		Water: 15 dps **NS**			

*AF* Abdominal Fat, *AG* Adrenal Glands, *CSC* Chronic Subordinate Colony housing, *CSDS* Chronic Social Defeat Stress, *CSS* Chronic Social Stress, *CMS* Chronic Mild Stress, *dps* days post-stress, *F* Females, *M* Males, *mps* month post stress, *NS* non-significant, *PG* pituitary gland, *S* Spleen, *SD/OC* social defeat/ overcrowding, *SV* seminal vesicles, *T* testicules, *TH* thymus, *wps* weeks post stress, ▲/▼ increase/decrease compared to control or baseline if not stated otherwise

## Central effects

The central nervous system and specifically the brain is the key to the perception and processing of physical and psychological stressors but also controls the individual’s behavioral reaction. Considering the limited amount and types of available long-term data, we first cover changes in brain structure, and follow up with molecular findings from the literature.

### Cellular effects

#### Neuronal cells

Neuronal activity in the brain 2.5 weeks after CSDS upon social interaction as a stimulus was previously assessed via c-Fos staining by Okamura and colleagues. They found a reduced activation in several regions including the hippocampus, nucleus accumbens (NAc), central amygdala (CeA), and medial prefrontal cortex (mPFC). Moreover, they found increased synchronicity between these brain regions, which are linked to symptoms of depression, fear, and memory impairment (Okamura et al. 2022).

The hippocampus plays an important role in learning and memory (Young et al. 1997), but also shapes stress response and emotions along with the hypothalamus and the amygdala (Fanselow and Dong 2010). Two studies evaluated adult neurogenesis in the hippocampus four weeks after CSDS and found opposing results: Chen and colleagues reported impaired synaptic maturation, reduced dendritic complexity and survival of adult-born dentate granule cells (DGCs) (Chen et al. 2016), while the study by Lagace and colleagues showed an increase of DGC survival in socially avoidant and thus susceptible mice (Lagace et al. 2010). Besides differences in the experimental protocol discussed in the more recent publication, it is also of note that Lagace and colleagues retested the social avoidance before sacrifice and based their classification on this later result. This may have led to the definition of another subgroup of animals in light of recent evidence of individual changes in social avoidance behavior longitudinally following CSDS (Okamura et al. 2022).

At the cortical level, the prefrontal cortex (PFC) is involved in emotional control by preventing the impact of high stress levels on the amygdala (Arnsten 2009). The medial prefrontal cortex (mPFC) regulates emotions, particularly anxiety, working memory, decision-making, and plays a key role in higher executive functions (Akirav and Maroun 2007; Patel et al. 2019). One study showed lowered spine density of excitatory neurons of the PFC one month post CSDS (Colyn et al. 2019). The amygdala integrates emotions, regulates emotional responses particularly to fear and stress, and is responsible for memory processing in interaction with the PFC and hippocampus (Tyng et al. 2017). The basolateral part of the amygdala (BLA) is critical for the formation and retrieval of conditioned fear memories (Likhtik and Paz 2015), and CSDS enhanced dendritic arborization in this important region one month after stress (Colyn et al. 2019). Previous studies in rodents already hypothesized that such structural alterations of the BLA are the consequence of diminished feedback control of the PFC over the amygdala (Jackson and Moghaddam 2001; Huang et al. 2020) and could explain elevated levels of anxiety or impair social behavior. In line with this theory, Okamura and colleagues observed a reduced correlation between the BLA and mPFC c-Fos expression two weeks after stress (Okamura et al. 2022).

Neurobiological dissection of depressive behavior must focus on anhedonia, the loss of feeling interest in and reward from pleasureful stimuli, as one of the key behavioral changes in patients but also mice upon chronic stressors (Slattery and Cryan 2017). The mesolimbic dopaminergic VTA-NAc circuit plays a critical integrative role in reward- and emotion-related behaviors (Nestler and Carlezon 2006). Moreover, it is also a prerequisite for the perception of emotional stimuli in general, but social status and the appraisal of threats from the social environment in particular (Berton et al. 2006; Krishnan et al. 2007). Consequently, short-term, but rarely long-term consequences have been studied extensively after chronic stress. Berton and colleagues reported increased c-Fos expression in the VTA and its target neurons of the NAc when exposed to a social partner four weeks post CSDS. This finding occurred in conjunction with a persistent transcriptional activation pattern in the NAc (Berton et al. 2006). Seemingly opposing results with a lower neuronal VTA activation in socially avoidant versus non-avoidant mice were seen by Okamura and colleagues at 2.5 weeks after CSDS (Okamura et al. 2022). One explanation could be the differing time points of these observations but also the study designs and differing comparisons between the treatment groups. This again highlights the demand for more long-term data with a focus on comparability of experimental designs. In an electrophysiological study in anesthetized animals, increased burst frequency of dopaminergic neurons in the VTA was detected three weeks after cessation of CSDS (Razzoli et al. 2011a). This confirmed and extended the ex vivo studies previously executed up to two weeks post CSDS (Krishnan et al. 2007). Together, these data demonstrate that chronic and stimulus-independent alterations of circuits, crucial for social interaction, motivation, and reward, are long-term determinants for stress susceptibility.

The primary motor cortex (M1) initiates and controls voluntary movements and contributes to motor learning. Although motor symptoms are known to occur in psychiatric disorders like major depression (Sobin and Sackeim 1997), M1 was previously not a region associated with stress effects. By in vivo imaging of M1, we detected a persistent reduction of dendritic spines and disturbed spine dynamics of excitatory neurons upon CSDS, which lasted up to 23 days (Gellner et al. 2022). This altered neuroplasticity was also reflected in impaired motor learning skills already mentioned in the behavioral section of this review. The proper balance of excitation and inhibition is a prerequisite for neuroplasticity and learning, and short-term alteration of inhibitory neuronal networks by chronic stress have been well-described (Duman et al. 2019), while data on long-term effects are lacking. We recently demonstrated a layer-dependent reduction of inhibitory parvalbumin or somatostatin positive interneurons in M1 five weeks after CSDS (Serradas et al. 2022).

From the limited data available, it can be debated but not resolved, whether stress-induced changes are a deficient response or a form of neuro-plastic adaptation, or both. Therefore, these structural effects need to be examined longitudinally after stress with the growing repertoire of in vivo methods.

#### Non-neuronal cells

Microglia and astrocytes are also known to respond rapidly to various stressors (Singh et al. 2011; Norden et al. 2016). Once activated, they can maintain neuro-inflammatory cascades and alter neuroplasticity that these cells regulate physiologically as part of the quad-partite synapse (Schafer et al. 2013) and pathologically in response to stress (Delpech et al. 2015; Bollinger and Wohleb 2019).

Primarily, microglia are known as the resident innate immune cells of the brain, typically primed or activated by a variety of pathogens (Hanisch and Kettenmann 2007), but also stressors (Jurgens and Johnson 2012; Bollinger and Wohleb 2019). Accordingly, microglia isolated from mice exposed to CSDS 24 days earlier exhibited a primed, pro-inflammatory mRNA signature. This was in line with sensitization to a lipopolysaccharide challenge with increased sickness behavior in stressed animals (Weber et al. 2019). Wohleb and colleagues thoroughly analyzed macrophage recruitment from the bloodstream to the brain, which occurs in parallel with microglial activation. They found macrophages increased in brain tissue at day eight but not 24 after CSDS. While their mRNA levels of pro-inflammatory Interleukin-1 beta and TNFalpha were elevated eight days but not 24 days post stress, the mRNA levels of IL-6, CD14, and CX3CR1 remained elevated until day 24 (Wohleb et al. 2014). This is consistent with the observation of another group, that pharmacological depletion of microglia throughout the CSDS phase prevented anxious behavior in the Light Dark Box Test two weeks post stress (Lehmann et al. 2019). Recently, we found morphological correlates of microglia activation in the motor cortex five weeks after the cessation of CSDS along with increased microglia-dendrite co-localization, supporting a link to impaired structural neuroplasticity in M1 (Gellner et al. 2022). Regarding the long-term astrocytic response to stress in mice, no data are available to our knowledge, apart from our finding of astrogliosis in the superficial primary motor cortex five weeks after CSDS (Gellner et al. 2022). We have discussed this as a potential consequence of the disturbed balance between excitation and inhibition mentioned earlier.

### Molecular effects

The molecular changes that underlie or accompany structural changes following chronic stress have rarely been elucidated from a long-term perspective. Dopaminergic signaling is crucial for the reward system mentioned earlier (Baik 2020). The function of the frontal cortex and the dorsal raphe nuclei is severely impaired by dopaminergic dysfunction after CSDS and has been discussed as a reason for long-term anhedonia (Venzala et al. 2013). The study by Venzala and colleagues also compared the chronic neurochemical effects of the CUMS model three to four weeks after its termination as well as four weeks after CSDS by determination of gamma-aminobutyric acid (GABA), glutamate, 5-HT, and dopamine levels in the PFC, raphe nuclei, and hippocampus. While both stress models resulted in decreased levels of the inhibitory neurotransmitter GABA and an increased ratio of excitatory to inhibitory neurotransmitters (glutamate/GABA) in the PFC, only socially stressed mice exhibited decreased levels of dopamine in this brain region. In contrast, the hippocampus showed no changes (Venzala et al. 2013). The dorsal raphe nuclei, recently described as highly relevant to appetitive and aversive memories and behaviors (Lin et al. 2020), displayed neurochemical effects that depended on the type of stressor: dopamine was decreased and glutamate increased after CSDS, whereas GABA decreased as a result of CUMS (Venzala et al. 2013). The authors thus contributed to understanding the mechanisms likely underlying the variable individual symptomatology found in depressive disorders.

Brain-derived neurotrophic factor (BDNF) plays a central role in regulating dopaminergic signaling in response to stress, the remodeling of dendrites in the hippocampus, the BLA (McEwen et al. 2015; Colyn et al. 2019) and the NAc (Berton et al. 2006; Krishnan et al. 2007). Moreover, BDNF also mediates long-term neural and behavioral plasticity in response to aversive social experiences. The VTA-NAc circuit is a crucial mediator of the response to CSDS, and several studies showed short-term effects with increased BDNF protein levels in the NAc induced by VTA activation (Berton et al. 2006; Krishnan et al. 2007). This effect in the NAc was detectable up to four weeks post CSDS (Berton et al. 2006). In contrast, CSDS resulted in downregulation of BDNF in the hippocampus, which was still evident four weeks afterward (Tsankova et al. 2006). Finally, BDNF and its precursor pro-BDNF are important players in dendritic growth and spine formation (Benarroch 2015). Thus, increased pro-BDNF levels in the amygdala triggered by re-exposure of a mouse to a one-time social defeat stress one month post CSDS fit well with the increased dendritic branching found in this brain region (Colyn et al. 2019).

An overview of the section on the cellular and molecular effects found over the long-term following chronic stress is given in Table 3.

**Table 3:** Cellular and molecular long-term effects in the brain after chronic stress

	Publication	Stress model	Time post-stress	Strain and sex	Cellular and molecular effects					
					Neuronal cells					Microglia/astrocytes
					Amygdala	Hippocampus	PFC	VTA/ NAc	Motor cortex	
Social stress	Berton et al. (2006)	CSDS (10 min/10 days)	4 weeks	Transgenic mice, M				c-Fos ▲ (VTA, NAc) BDNF ▲ (NAc)		
	Chen et al. (2016)	CSDS (10 min/10 days)	2–4 weeks	C57BL/6 J M		2 wps Dendritic length ▼ 2 – 4 wps Branch number ▼ 4 wps Adult-born DGCs survival ▼				
	Colyn et al. (2019)	CSDS (10 min/10 days)	4 weeks	C57BL/6 J M	Dendritic arborisation ▲ pro-BDNF▲		Spine density ▼			
	Gellner et al. (2022)	CSDS (5 min/10 days)	11–23 days	Thy1-GFP M					Spine density **▼**	Reactive morphology ▲ Number of astrocytes ▲ (all in M1)
	Lagace et al. (2010)	CSDS (10 days)	4 weeks	nestin-GFP transgenic mice, M		DGCs survival ▲				
	Lehmann et al. (2019)	CSDS (5 min/14 days)	2 weeks	C57BL/6 J M						▼ in cortical and subcortical regions
	Okamura et al. (2022)	CSDS (10 min/7 days)	2 weeks	Balb/c M	c-Fos ▼	c-Fos ▼	c-Fos ▼	c-Fos ▼ (VTA, only in avoidant mice)		
	Razzoli et al. (2011a)	CSDS (10 min/7 days)	3 weeks	C57BL/6 J M				Activity ▲		
	Razzoli et al. (2011d)	CSDS (10 min/10 days)	4 weeks	TrkB.T1 mice/WT, M		**NS**				
	Serradas et al. (2022)	CSDS (5 min/10 days)	5 weeks	C57BL/6 J M					staining of PV and SST positive interneurons ▼	
	Tsankova et al. (2006)	CSDS (10 min/10 days)	4 weeks	C57BL/6 J M		BDNF **▼**				
	Venzala et al., (2013)	CSDS + CMS (10 days/6 weeks)	4 weeks	C57BL/6 J M		GABA, Glut, Dopamine, 5-HT **NS** (CSDS, CMS)	GABA, GABA/Glut ratio ▼ (CSDS, CMS) Dopamine ▼ (CSDS) 5-HT **NS**			
	Weber et al. (2019)	CSDS (10 min/10 days)	24 days	C57BL/6 J M						Proinflammatory mRNA signature (microglia) ▲
	Wohleb et al. (2014)	CSDS (2 h/6 days)	8–24 days	C57BL/6 J M						Proinflammatory mRNA signature (microgl., 24 dps) ▲ Macrophages ▲ (8 dps)

*BDNF* Brain Derived Neurotrophic Factor, *CMS* Chronic Mild Stress, *CSDS* Chronic Social Defeat Stress, *DGCs* Dentate Granule Cells, *dps* days post stress, *Glut* Glutamate, *5-HT* Serotonine, *M1* primary motor cortex, *NAc* Nucleus Accumbens, *NS* non-significant, *PFC* Pre-Frontal Cortex, *PV* Parvalbumin, *SST* Somatostatin, *VTA* Ventral Tegmental Area, *wps* week post-stress, ▲/▼ increase/decrease compared to control or baseline if not stated otherwise

## Conclusion

We collected data on long-term stress effects in mice, which we defined as at least one week or later after cessation of the stressor. To date, there are only a few studies that have looked at long-term outcomes. Therefore, we had to carefully search for co-studies in publications that primarily reported short-term observations. The comparatively small number of studies meeting our requirements had several consequences: we refrained from further differentiation or clustering of stress protocols, their durations, or mouse strains, nor did we distinguish between outcomes that were repeatedly measured in both short and long term or tested only in the long-term. We can conclude from the limited data available that short-term behavior does not automatically equal long-term behavior, which has already been demonstrated in some of the studies included in this review. From a translational perspective, stress-related diseases in humans are often diagnosed at an advanced or even chronic state, with earlier symptoms being assessed only retrospectively. Of course, it is difficult to accurately match the ages of humans and mice or the equivalents of certain time spans e.g., for the definition of “long-term”. Compared to animal research, in clinical studies, individual characteristics, such as the duration of the disease and the duration and/or persistence of the stressor, cannot be perfectly controlled before assessing (neuro)biological outcomes. This is especially true for information from brain tissue, which is usually obtained from a specific subset of patients, e.g., those who died by suicide. In contrast to human studies, mouse models allow age ranges, duration of conditions, and the type of stressor to be specifically combined, as well as a broad choice of post hoc assessments. Therefore, these animal models provide valuable and multifaceted information about subpopulations of patients. Consequently, translation of mouse data to humans depends on a critical stratification of human data.

Women have a much higher prevalence of various chronic stress-related illnesses, such as major depression, anxiety, or post-traumatic stress disorder. As a general development in biomedical animal research, studies with female rodents need to increase drastically. This will also require efforts to develop appropriate models that account for differences between the sexes. Further emphasis needs to be placed on mouse strain selection, as has been repeatedly mentioned in this review, as innate and stress-induced behavior can differ in short and long term.

Overall, mouse studies of chronic stress need to extend beyond the early days after stress. With a growing body of long-term data, further division of this phase, e.g., into early and late long-term phases, will be possible with regards to translation. Lastly, the discoverability of long-term data in the literature needs to be improved by highlighting these findings in titles, abstracts, and keywords.

## Data Availability

Data sharing not applicable to this article as no datasets were generated or analysed.
